# Prevalence of Human Papillomavirus Among Chinese Han and Mongols Minority Women in Inner Mongolia, China: Reflected by Self-Collected Samples in CHIMUST

**DOI:** 10.3389/fpubh.2022.840879

**Published:** 2022-05-25

**Authors:** Chunlei Guo, Hui Du, Xinfeng Qu, Xianzhi Duan, Jingran Li, Ruizhen Li, Hua Jin, Chun Wang, Chao Zhao, Juncui Bao, Hongxue Luo, Lihui Wei, J. L. Belinson, Ruifang Wu

**Affiliations:** ^1^Department of Obstetrics and Gynecology, Peking University Shenzhen Hospital, Shenzhen, China; ^2^Institute of Obstetrics and Gynecology, Shenzhen Peking University-Hong Kong University of Science and Technology (PKU-HKUST) Medical Center, Shenzhen, China; ^3^Shenzhen Key Laboratory on Technology for Early Diagnosis of Major Gynecologic Diseases, Peking University Shenzhen Hospital, Shenzhen, China; ^4^Capital Medical University Beijing Tongren Hospital, Beijing, China; ^5^Peking University People's Hospital, Beijing, China; ^6^Wushenqi People's Hospital, Inner Mongolia, China; ^7^Preventive Oncology International, Inc., and the Cleveland Clinic, Cleveland, OH, United States

**Keywords:** human papillomavirus, Chinese Han, Mongols minority, type-specific prevalence, cervical intraepithelial neoplasia, distribution

## Abstract

**Background:**

The disparities of hr-HPV infection among races/ethnicities have not been fully discussed. This study aimed to investigate the difference of hr-HPV infection between Chinese Han and Mongols minority women in Inner Mongolia.

**Methods:**

Genotyping and histopathology data of Chinese Han and Mongols minority women in Inner Mongolia from Chinese Multi-Center Screening Trial were used to analyze the hr-HPV prevalence, and type-specific distribution in abnormal pathology results.

**Results:**

The hr-HPV infection rates of Han women was 15.9% while of Mongols was 21.6% (*P* < 0.001). The most prevalent genotypes in Han women were ranked as HPV-16,−52,−18/-58,−31/-39, and−59 while in Mongols were−16,−31,−58,−18 and−52. When analyzing the age-specific of hr-HPV infection, two peaks were found at age of 40–44 (20.5%) and 55–59 (23.5%) years in Han women while three peaks were observed at age of 30–34 (22.1%), 45–49 (22.9%), and 55–59 (31.8%) years, respectively, in Mongols. HPV-16 accounting for 62.5 and 53.8% of the CINII+ in Han and Mongols, respectively.

**Conclusion:**

The prevalence of hr-HPV was significantly different between the Han and Mongols minority women in Inner Mongolia, races/ethnicities background should be taken into consideration for the refinement of cervical cancer screening strategies and vaccine implementation in China.

## Introduction

Invasive cervical cancer (ICC) is one of the top threats to women's health in many regions of the world, particularly in low- and middle-income countries (LMICs) (1). In China, over 100,000 women were newly diagnosed with ICC and ~50,000 dies of this preventable malignancy per year (2). Persistent high-risk human papillomavirus (hr-HPV) infection plays a vital role in triggering the development of ICC and cervical intraepithelial neoplasia (CIN) (3, 4). Cervical screening using exfoliative cytology has effectively reduced the morbidity and mortality of this disease over the past decades (5, 6) in the medically well-served high income countries. However, there is a strategical shift from cytology-based to molecular-based (HPV) screening and further refined using risk-based strategies (primarily around HPV genotyping) (7–9). These changes have amplified the potential for using self-collection of cervical-vaginal samples for cervical cancer screening (10, 11).

Numerous studies have demonstrated that a significant geographical variation exists in the prevalence and age-distribution of HPV infection. For example, HPV prevalence varies from 2.8% in North American women aged 55–64 years to 50.5% in Eastern African women aged 25-34 years (12); and the carcinogenic potential varies greatly by genotype. HPV-16 and−18 together are reported to be responsible for 71% of ICC globally (13) and 84% in China (14). Even in mainland China, according to our prior study, there were disparities in the prevalence and type-specific distribution of HPV in different provinces (15). The causes for these differences are still unclear, although socioeconomic factors and behavioral risk factors have been suggested as the most possible explanations (16), biological and genetic factors should not be ignored.

The Inner Mongolia Minority Autonomous Region (Inner Mongolia), located on the northern border of China, is a multi-ethnic region with an estimated over 4 million Mongolian. This is ranked second to the Han according to the seventh census report published online by the National Bureau of Statistics. People of different ethnicities in Inner Mongolia have lived together over vast areas while some still live in individual concentrated communities. To better understand the prevalence and type-specific distribution of hr-HPV among the women in Inner Mongolia, we analyzed the data from the Chinese Multi-Center Screening Trial among Chinese Han and Mongol women.

## Materials and Methods

### Study Population

This Chinese Multi-Center Screening Trial (CHIMUST) (Registration number: ChiCTR-EOC-16008456) is a multi-center, cross-sectional, population-based cervical cancer screening trial conducted between Aug 2016 and Jan 2018 at 15 sites located in 6 provinces as Beijing (the capital city), Inner Mongolia, Hebei, Hubei, Jiangxi, and Guangdong in mainland China. The inclusion criteria were: 30–59 years of age, sex exposed and no cervical cancer screening in the past 3 years. The exclusion criteria were: in pregnant, or had pelvic radiation and hysterectomy history. A total of 10,885 women were enrolled. The protocol of this trial was approved by the Institutional Review Board (IRB) of Peking University Shenzhen Hospital (IRB: PUSH2016001) and Cleveland Clinic Institutional Review Board (IRB: 15–1549). All participants signed an informed consent document before enrollment.

All participants were required to provide a self-collected cervical-vaginal sample when they arrived at hospital, self-sampling methods can be obtained from researcher or a special brochure printed for the research. Then a physician-collected endocervical sample was also obtained. The brush with self-collected sample was first applied onto a solid media transport card and then be placed in PreservCyt solution, The physician collected samples were obtained by a trained gynecologists by using a “broom” sampler and then placed in 20 mL of Thin-Prep PreservCyt Solution. All samples were tested with the PCR-based high-risk HPV assays: Cobas4800 (Roche) and SeqHPV (BGI-Shenzhen, China). The physician-collected samples were also processed for cytology and interpreted by cyto-pathologists from Peking University Shenzhen Hospital (PUSH) with facilitation of the Hologic I2 Imager (computer assisted cytology). Women tested positive of hr-HPV on either or both Cobas4800 or/and SeqHPV for either or both of self- or/and direct-collected samples were referred for colposcopy using the quadrant-based POI (Preventive Oncology International Inc.) protocol of directed and random biopsies plus ECC (17). The materials and methods for the entire CHIMUST trial has been detailed in a prior publication (18).

**Figure 1:**
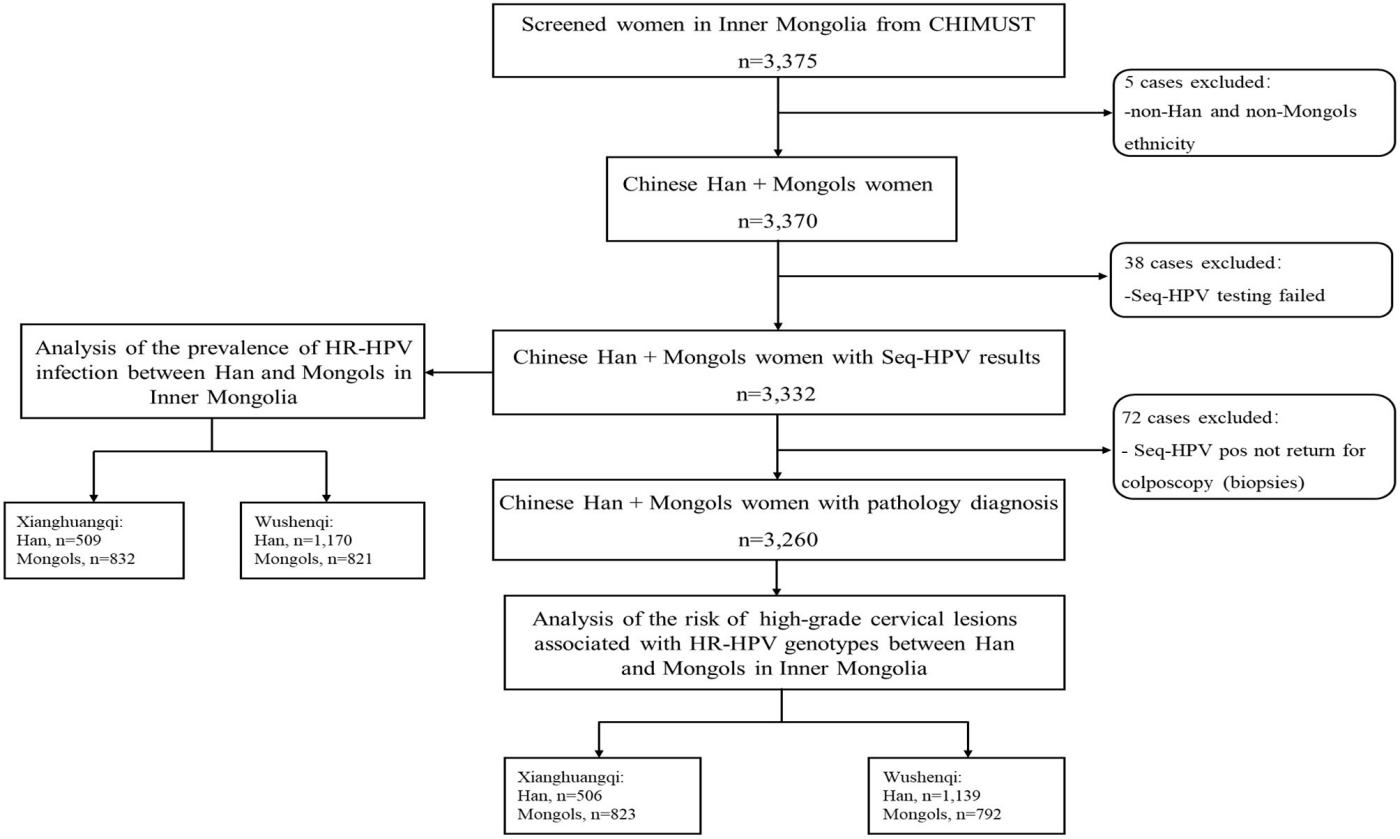
Study population.

For this manuscript, the data of CHIMUST related to the 3,375 women aged 30–59 years enrolled in Inner Mongolia was analyzed. Participants were not included in the analysis for the prevalence of hr-HPV infection in Han and Mongols if they were demographically neither Han or Mongols or failed HPV testing for SeqHPV due to inadequate DNA or failed PCR. Women positive of hr-HPV but not returned for colposcopy were excluded from the evaluation of the distribution of individual hr-HPV genotypes among the women with pathological abnormalities. The project was conducted at sites in two counties, Xianghuangqi and Wushenqi, in Inner Mongolia. Data was also analyzed by regions since the distance between these two subregions is nearly 600 kilometers (Figure 1).

### Study Methods

#### SeqHPV Assay

The SeqHPV is a NGS (next-generation sequencing) based assay for hr-HPV, which uses multiplex PCR to amplify DNA and next generation sequencing to identify HPV genotypes. It amplifies the HPV L1 gene and human β-globin gene (HBB) (Housekeeping gene to identify false negatives caused by inadequate DNA or failed PCR) of cervical exfoliated cells in the sample. It is a high-throughput technology that can identify 14 hr-HPV genotypes (HPV-16,−18,−31,−33,−35,−39,−45,−51,−52,−56,−58,−59,−66, and−68) (19). All procedures were carried out in strict accordance with the working manual of the testing technology and the guidelines for the companion kit.

#### Pathological Diagnosis of Biopsy

The colposcopy protocol used in this trial was done by quadrant. Based on the protocol, direct biopsies were performed on where lesion was visible and random biopsies were performed at the squamocolumnar junction in the normal quadrants. All colposcopy patients had an endocervical curettage (ECC) (17). Cytology were not referred for study colposcopists and the pathologists who review the pathology slides were blinded of the HPV results. Pathological diagnoses were reported as negative (for intraepithelial lesion/malignancy), cervical intraepithelial neoplasia (CIN) grade I, II, and III, adenocarcinoma, and squamous cell carcinoma. The highest grade among the multiple biopsies from each quadrant and the ECC was recorded as the final diagnosis.

#### Statistical Analysis

To minimize the possibility of selection bias being introduced by deleting cases without histologic diagnose, women with SeqHPV testing results were included in the analysis of the agreement between self-and physician-collected sample and the prevalence and type-specific distribution of hr-HPV in Han and Mongols minority. The race/ethnicity of the participants were self-reported. Consistency between self-and physician- collected sample was measured by absolute agreement and Kappa statistics (Cohen's Kappa). Pearson's Chi-squared test with two-tailed was performed to calculate differences at a significant level of 0.05 to compare the prevalence and distribution of hr-HPV infection between Han and Mongols minority. The prevalence of hr-HPV genotypes was also calculated separately for 6 age groups (30–34, 35–39, 40–44, 45–49, 50–54, and 55–59 years) race/ethnicity and by region. Women of the two ethnicities with both SeqHPV and histologic diagnose were included in the analysis of the prevalence of type-specific hr-HPV infection in abnormal pathology results. The 95% confidence interval (CI) was established using a binomial distribution analysis. SPSS v.24.0 software (IBM, Armonk, NY, USA) was used for all data analysis in this study.

## Results

### Sample Information

A total of 3,375 women aged 30–59 years were enrolled in Inner Mongolia. Of these, 5 who were non-Han and non-Mongols and 38 who failed SeqHPV testing for inadequate DNA/ failed PCR were excluded, leaving 3,332 women with SeqHPV testing results for the analysis of the prevalence and distribution of hr-HPV between Han and Mongols. The mean age of the analyzed women was 43.8 ± 7.7 years (median age 44 years and range 30–59 years). The overall prevalence of Hr-HPV was 18.7% (95% CI 17.4–20.1%) for the self-collected HPV testing, with the most prevalent genotypes being HPV-16 (3.8%), −31(2.6%), −58 (2.3%), −18 (2.1%), and −52 (2.0%) in respective orders. After excluded 72 women who did not return for colposcopy (no histologic diagnoses) 3,260 women with a mean age 43.9 ± 7.6 years (median age 44 years and range 30–59 years) were included in the analysis of the risk of having histologically confirmed high-grade disease relative to HPV genotypes. Among these 3,260 women, 96.2% (3,135/3,260) were pathologically normal, 2.5% (83/3,260) had CINI, 1.3% (42/3,260) had CINII+.

### Consistency of the SeqHPV Assays in Self-Collected and Physician-Collected Sample

Among the 3,332 women included for the analysis, hr-HPV prevalence was 18.7% (624/3,332) for self-collected testing while 16.8% (559/3,332) for physician-collected testing, with 530 (15.9%, 530/3,332) women positive of hr-HPV on SeqHPV for both self-and physician-collected samples. The concordance of HPV positive between physician-collected and self-collected samples was 81.2% (530/653), showing an “near perfect” consistency in SeqHPV detection between self-and physician-collected samples (Kappa = 0.87, 95% CI 0.85–0.90, *P* < 0.001).

### Prevalence and Type-Specific Distribution of hr-HPV Infection in Self-Collected Samples Between Han and Mongols

The hr-HPV prevalence in Mongol women was 21.6% (95% CI 19.6%-23.6%), significantly higher than that in Han women, which was 15.9% (95% CI 14.2–17.7%) (χ^2^ = 17.748, *P* < 0.001). The most prevalent genotypes in Han women were HPV-16 (3.2%),−52(2.1%),−18 and−58 (both 1.8%),−31 and−39 (both 1.6%), and−59 (1.3%) in order, while those in Mongols were−16 (4.5%),−31 (3.7%),−58 (2.7%),−18 (2.4%) and−52 (1.9%) (Table 1).

**Table 1:** The prevalence and type-specific distribution of hr-HPV between Han and Mongols women (*n*; %).

**HPV genotypes**	**Han** **(*n* = 1,679)**	**Mongols** **(*n* = 1,653)**	**c^**2**^ value^**a**^**	***P-*value**
HR-HPV pos^b^	267; 15.9	357; 21.6	12.209	<0.001
HPV-16	53; 3.2	74; 4.5	1.645	0.200
HPV-18	30; 1.8	40; 2.4	2.137	0.144
HPV-31	27; 1.6	61; 3.7	8.565	<0.05
HPV-33	15; 0.9	19; 1.1	0.105	0.746
HPV-35	16; 1.0	25; 1.5	0.699	0.403
HPV-39	27; 1.6	29; 1.8	0.003	0.957
HPV-45	9; 0.5	27; 1.6	2.813	0.093
HPV-51	15; 0.9	28; 1.7	1.832	0.176
HPV-52	35; 2.1	32; 1.9	0.042	0.838
HPV-56	16; 1.0	16; 1.0	0.002	0.968
HPV-58	30; 1.8	45; 2.7	4.81	<0.05
HPV-59	21; 1.3	16; 1.0	0.019	0.891
HPV-66	12; 0.7	13; 0.8	0.001	0.971
HPV-68	13; 0.8	24; 1.5	2.781	0.095

a *Pearson's Chi-square test was performed to compare the prevalence of HPV infection between Han and Mongols in two regions of Inner Mongolia*.

b *On account of multi-infection, the number of HR-HPV pos may less than the sum of each HPV genotype*.

In Xianghuangqi (XHQ), the Hr-HPV infection rates between Han- XHQ and Mongols- XHQ women was 11.4% (95% CI 8.6–14.2%) and 19.6% (95% CI 16.9–22.3%), respectively, significantly different with each other (χ^2^ = 15.414, *P* < 0.001). In Wushenqi (WSQ), the hr-HPV infection rates between Han-WSQ and Mongols-WSQ women was 17.9% (95% CI 15.7–20.1%) and 23.6% (95% CI 20.7–26.5%), respectively, and the difference was also statistically significant (χ^2^ = 9.937, *P* = 0.002; Supplementary Materials).

### Prevalence of Age-Specific hr-HPV Infection on Self-Collected Sample Between Han and Mongols

**Figure 2:**
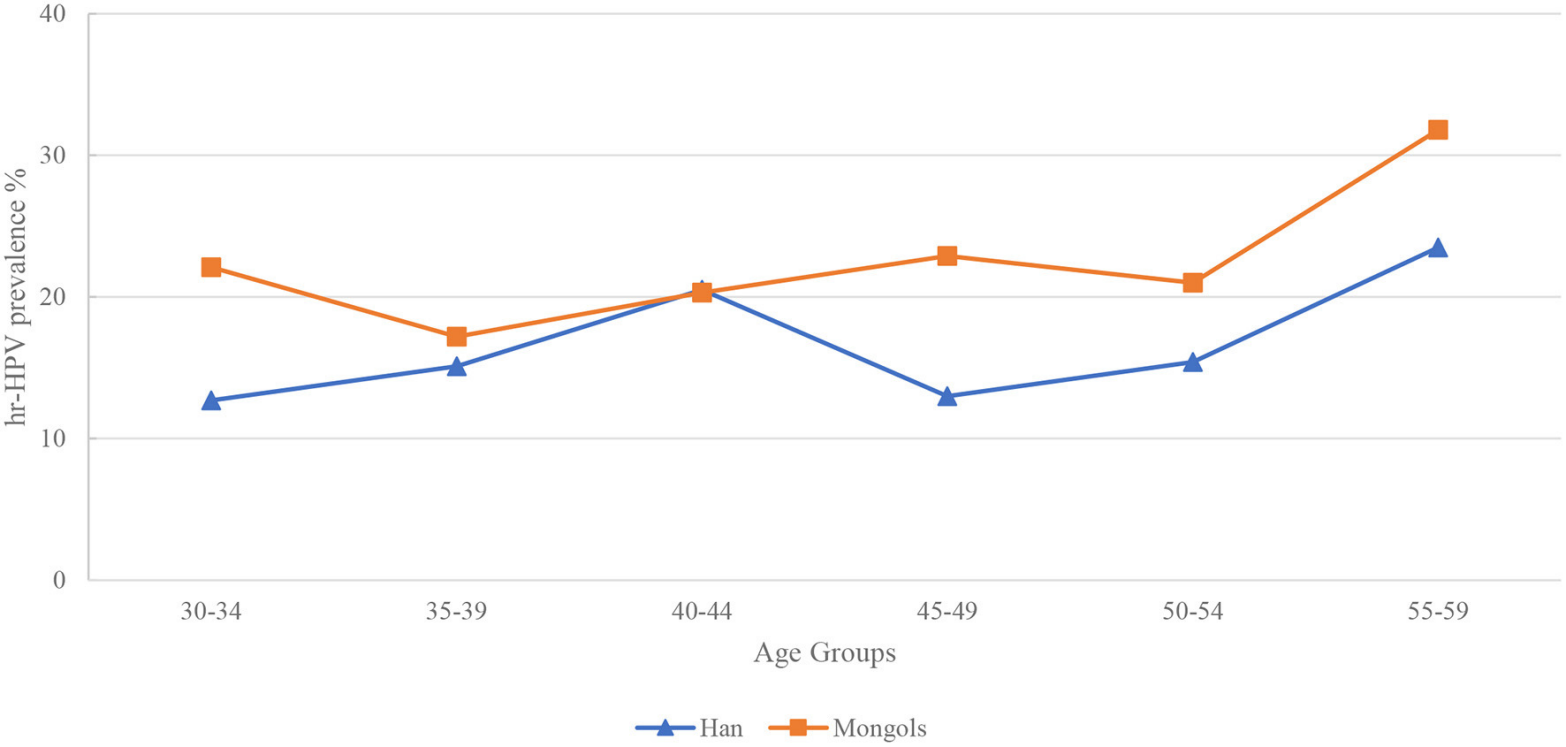
Age-distribution of Hr-HPV prevalence, stratified by ethnicity.

All participants were divided into 6 age groups (30–34, 35–39, 40–44, 45–49, 50–54, and 55–59 years). Two peaks of hr-HPV infection were found at age 40–44 (20.5%) and 55–59 (23.5%) years in Han women. In Mongol minority women, the age-specific hr-HPV infection curve is just like a “W,” with three peaks at age 30–34 (22.1%), 45–49 (22.9%) and 55–59 (31.8%) years, respectively. Interestingly, a highest prevalence at age 55–59 years was observed in two ethnicities prevalence curves (Figure 2).

The prevalence of hr-HPV in Han-XHQ women raised steadily from age 30–34 years and reached the peak at age 40–44 (14.3%) years then dropped down and had a less pronounced second peak at 50–54 (11.2%) years. In contrast to Han-WSQ and Mongols minority, Han-XHQ women had the lowest prevalence of hr-HPV at age 55–59 years (Supplementary Materials).

### Prevalence of Type-Specific hr-HPV Infection in Abnormal Pathology Diagnoses in Han and Mongols

The diagnosed CINI in Mongols women was rated as 3.3% (54/1,615) per the screened Mongols women, significantly higher than the same rate of 1.8% (29/1,645) in Han Women (χ^2^ = 8.207, *P* = 0.004). However, no significant difference was observed between the two ethnicities for the diagnosed CINII+ (1.0 vs. 1.6%, χ^2^ = 2.602, *P* = 0.107). In Han women with CINI, HPV-52 together with−58 and−59 (all 13.8%), were the most detectable genotypes, while in Mongols, HPV-16 and−31 (both 16.7%) were the dominant genotypes. In high grade disease (CINII+) HPV-16 was dominant among the 14 genotypes, representing 62.5 and 53.8% of the diagnosed cases in Han and Mongols, respectively. Particularly, HPV-52 and−59, the most prevalent genotypes in Han women, were not found to be related with CINII+ cases (Table 2).

**Table 2:** Prevalence of type-specific Hr-HPV infection in abnormal pathology diagnoses in Han and Mongols (*n*; %).

**Genotypes**	**CINI**		**CINII+**	
	**Han (*n* = 29)**	**Mongols (*n* = 54)**	**Han (*n* = 16)**	**Mongols (*n* = 26)**
Hr-HPV pos^a^	22; 75.9	43; 79.6	16; 100.0	23; 88.5
HPV-16	2; 6.9	9; 16.7	10; 62.5	14; 53.8
HPV-18	3; 10.3	3; 5.6	1; 6.3	2; 7.7
HPV-31	1; 3.4	9; 16.7	2; 12.5	2; 7.7
HPV-33	3; 10.3	1; 1.9	1; 6.3	3; 11.5
HPV-35	0; 0.0	4; 7.4	0; 0.0	1; 3.8
HPV-39	2; 6.9	3; 5.6	2; 12.5	1; 3.8
HPV-45	0; 0.0	1; 1.9	0; 0.0	2; 7.7
HPV-51	3; 10.3	3; 5.6	1; 6.3	1; 3.8
HPV-52	4; 13.8	4; 7.4	0; 0.0	1; 3.8
HPV-56	0; 0.0	1; 1.9	1; 6.3	1; 3.8
HPV-58	4; 13.8	8; 14.8	3; 18.8	1; 3.8
HPV-59	4;13.8	1; 1.9	0; 0.0	1; 3.8
HPV-66	0; 0.0	3; 5.6	0; 0.0	0; 0.0
HPV-68	2; 6.9	1; 1.9	0; 0.0	3; 11.5

a *On account of multi-infection, the number of Hr-HPV pos may less than the sum of each HPV genotype*.

## Discussion

To the best of our knowledge, this is the first epidemiologic study of HPV infection based self-collected samples HPV testing, and also the first one to compare HPV and CIN prevalence among Chinese Han and Mongol minorities in Inner Mongolia. The analysis was conducted based on data of CHIMUST because all the participants contributed two cervical-vaginal specimens, one was collected by participant-self under the instruction of printed guide (self-collected), the other was collected by trained gynecologists (physician-collected), the concordance between self-collected and physician-collected sample in detecting hr-HPV on SeqHPV was “near perfect” in Kappa terms (Kappa = 0.87, *P* < 0.001). Exfoliative cytology had been the foundation of worldwide cervical screening strategy for a long term. It works with its modest sensitivity and high specificity in detection of pre-cancers. However, since the sensitivity and negative prediction value determinant to the efficiency of a population screening project for prevention of cervical cancer, a shift of primary screening from cytology to hr-HPV testing in screening strategy occurred, with HPV genotyping further development to risk-based screening and management algorithms (8, 20). The negative predictive value of HPV testing has been a significant driver of this change since baseline HPV-negative women have a significantly lower cumulative incidence of CINIII+ at 48 months than cytology-negative women (21, 22). In addition, the effectiveness of self-collection with molecular screening will make it easier in the future to reach women in LMICs who were not well-served by cytology-based strategies due to both human as well as financial constraints (10).

In our trial the overall prevalence of hr-HPV infection in Inner Mongolia was 18.7% (95% CI 17.4–20.1%), higher than the rate of 14.5% in the same region reported by Wang et al. (23). This is likely secondary to the self-sampling used in our study. The prevalence of hr-HPV infection in our study population was consistently higher among the Mongolian women in each of the two counties as well as overall. This suggests that race/ethnicity rather than the geographic difference is the important co-factor for hr-HPV infection which is consistent with the conclusion of Baloch et al. (24).

Notably, HPV-16 was the most prevalent genotype in the overall study population or stratified by region or ethnicity. HPV-16 can be classified into four main evolutionary-derived variant lineages and nine sub-lineages: A1–4, B1–2, C, and D1–3 (25). By using high-throughput HPV whole-genome sequencing, Mirabello et al. studied the variant lineage risk in over 3,200 HPV16-infected women from Kaiser Permanente Northern California (KPNC). They found an association between race/ethnicity and infection with specific HPV16 variant lineages, and the risk of pre-cancer and cancer for specific HPV16 variant lineages. Asian women had an increased risk of CINIII+ associated with the A4 variants while white women infected with an A1/A2 variant had an increased risk of CINIII+ (26). The specific mechanisms that favor HPV-16 remain a mystery to be explored, but viral genetic variation may partly explain its unique prevalence and carcinogenic properties. Besides HPV-16, the type-specific distribution between Han and Mongols revealed HPV-18,−31,−52,−58, and−59 were commonly seen among the 5 most prevalent genotypes, with variable ranking, which was consistent with prior study (15). Moreover, HPV-39 ranked in the top group in Han, as well as Mongols-WSQ. Since both HPV-39 and−59 are not covered by any type of HPV vaccine (bivalent/quadrivalent/nine-valent vaccine), the infections with these two genotypes in Inner Mongolia are of concern.

Evidence on the prevalence of cervical hr-HPV infection across age group has been discussed in previous studies, with young women (<25 years) tending to have a higher incidence of hr-HPV infection than women in the other age groups (27, 28). Nevertheless, the updated guidelines recommended that cervical cancer screening begin at age 25 years, because the burden of cervical cancer among women ages 20 to 24 years is far less than that among women aged 25 to 29 years (29). In this manuscript, the enrolled women were ages 30–59 years, so we can not report the prevalence of hr-HPV among women aged <30 years, but other interesting age-related data were observed. The highest incidence of hr-HPV infection was observed among women aged 55–59 years (except for Han-XHQ women), very different from the previous studies (12). The reasons for this upsurge among the older age group was unclear, but declining immunity in the premenopausal and postmenopausal women resulting in a weakened ability to eliminate HPV infections is believed to be a possible reason (28). However, certain studies have indicated an increasing burden of human immunodeficiency virus (HIV)/AIDS among older adults, and unprotected sexual exposures were considered as possible explanations (30). Although we did not test for HIV, we are unaware of any HIV prevalence in our study populations.

No statistical differences were observed in histologic diagnosis between Han and Mongols. Our prior study had demonstrated the paramount role of HPV-16,−52, and 58 attributable to CINII+, but the majority of the study population were Han, the Mongol minority was <1% (31). Another meta-analysis on prevalence and attribution of HPV-52 and HPV-58 in cervical neoplasia worldwide indicated that these two genotypes shared a higher prevalence and attribution among cervical intraepithelial neoplasia in Eastern Asia; and the attribution of HPV-58 to invasive cervical cancer was nearly 2-fold higher than that of HPV-52 (32). In this study, HPV-52 was not a commonly seen genotype in CINII+ in either ethnicity and may indicate diminished carcinogenicity. HPV-58 was one of the most prevalent genotypes after HPV-16 in CINII+ in Han, while the proportion in Mongols was low. And regardless of the lower prevalence in both the two ethnicities, the high frequency of HPV-33 for CINII+ represent a relatively higher risk of pathogenicity than many other genotypes. He et al. did a polymorphism analysis of HPV-33/58 in Southwest China and identified some prevalent mutations that could have enhanced viral adaptability to the environment and increased the risk of carcinogenesis (33). We believe the differences may suggest that race/ethnicity and geographical background should be considered in modeling HPV carcinogenesis.

A major strength of this study is that it is a component of a large well-organized screening program. In addition, the study protocol included available technologies for screening and analysis as well as the uniform requirements for colposcopy referral, and the standard POI protocol for colposcopy/biopsy.

However, there are several limitations of this study. There was no data on the use of vaccination, occupations, smoking history, and number of sexual partners. Despite that, most enrolled women in the population were likely unvaccinated against HPV given that HPV vaccines have not been incorporated into our National Immunization Program yet, thus the coverage is low to non-existent. In addition, there might be some verification bias since women with a result of HPV negative were referred for routine screening.

In summary, we have characterized the prevalence and distribution of HPV infection in self-collected samples between the Han and Mongol minorities in Inner Mongolia, which demonstrated race/ethnicity differences in the prevalence of hr-HPV and their hr-HPV type-specific distribution. Given that China is a multi-ethnic country, the present study provided guidance for the refinement of cervical cancer screening strategies and vaccine implementation in China.

## Data Availability Statement

The original contributions presented in the study are included in the article/Supplementary Material, further inquiries can be directed to the corresponding author/s.

## Ethics Statement

The protocol of this trial was approved by the Institutional Review Board (IRB) of Peking University Shenzhen Hospital (IRB: PUSH2016001) and Cleveland Clinic Institutional Review Board (IRB:15–1549). All participants signed an informed consent document before enrollment. The patients/participants provided their written informed consent to participate in this study.

## Author Contributions

RW, LW, and JLB were involved in the conception of the study and obtaining ethical approval. CG, HD, and XQ analyzed and interpreted the patient data and drafted the manuscript. XD, JL, HJ, CW, CZ, JB, and HL selected participants and collected the data. All authors have contributed significantly to the final article and have approved it.

## Funding

The study was supported by Shenzhen High-level Hospital Construction Fund (YBH2019-260), Shenzhen Key Medical Discipline Construction Fund (Grant No. SZXK027), and Sanming Project of Medicine in Shenzhen (Grant No. SZSM202011016).

## Conflict of Interest

JB was employed by Preventive Oncology International, Inc., and the Cleveland Clinic. The remaining authors declare that the research was conducted in the absence of any commercial or financial relationships that could be construed as a potential conflict of interest.

## Publisher's Note

All claims expressed in this article are solely those of the authors and do not necessarily represent those of their affiliated organizations, or those of the publisher, the editors and the reviewers. Any product that may be evaluated in this article, or claim that may be made by its manufacturer, is not guaranteed or endorsed by the publisher.

## Abbreviations

CHIMUST: Chinese multi-center screening trial
hr-HPV: high-risk human papillomavirus
CIN: Cervical intraepithelial neoplasia
XHQ: Xianghuangqi
WSQ: Wushenqi
CI: Confidence interval.
