# Supplementary material for: Prevalence of Human Papillomavirus Among Chinese Han and Mongols Minority Women in Inner Mongolia, China: Reflected by Self-Collected Samples in CHIMUST
Source: Front Public Health. 2022 May 25;10:840879. doi: 10.3389/fpubh.2022.840879 (PMC9174663; doi:10.3389/fpubh.2022.840879)
Supplement: Supplementary file 1 [file Table_1.DOCX]

Table S1. Prevalence of type-specific Hr-HPV infection between Han and Mongols in Xianghuangqi and Wushenqi (n; %)

| HPV Genotypes | Xianghuangqi | | χ^2^ value^a^ | *P* value | Wushenqi | | χ^2^ value^a^ | *P* value |
| --- | --- | --- | --- | --- | --- | --- | --- | --- |
|  | Han (n=509) | Mongols (n=832) |  |  | Han (n=1,170) | Mongols (n=821) |  |  |
| Hr-HPV pos^b^ | 58; 11.4% | 163; 19.6% | 15.414 | ＜0.001 | 209; 17.9% | 194; 23.6% | 9.937 | 0.002 |
| HPV-16 | 10; 2.0% | 38; 4.6% | 6.198 | ＜0.05 | 43; 3.7% | 36; 4.4% | 0.638 | 0.452 |
| HPV-18 | 8; 1.6% | 18; 2.2% | 0.582 | 0.446 | 22; 1.9% | 22; 2.7% | 1.426 | 0.232 |
| HPV-31 | 9; 1.8% | 27; 3.2% | 2.637 | 0.104 | 18; 1.5% | 34; 4.1% | 12.850 | ＜0.001 |
| HPV-33 | 1; 0.2% | 7; 0.8% | 2.215 | 0.137 | 14; 1.2% | 12; 1.5% | 0.263 | 0.608 |
| HPV-35 | 1; 0.2% | 12; 1.4% | 5.106 | ＜0.05 | 15; 1.3% | 13; 1.6% | 0.316 | 0.574 |
| HPV-39 | 7; 1.4% | 12; 1.4% | 0.010 | 0.920 | 20; 1.7% | 17; 2.1% | 0.345 | 0.557 |
| HPV-45 | 1; 0.2% | 6; 0.7% | 1.674 | 0.196 | 8; 0.7% | 12; 1.5% | 2.936 | 0.087 |
| HPV-51 | 5; 1.0% | 14; 1.7% | 1.109 | 0.292 | 10; 0.9% | 14; 1.7% | 2.931 | 0.087 |
| HPV-52 | 6; 1.2% | 12; 1.4% | 0.166 | 0.684 | 29; 2.5% | 20; 2.4% | 0.004 | 0.952 |
| HPV-56 | 2; 0.4% | 8; 1.0% | 1.380 | 0.240 | 14; 1.2% | 8; 1.0% | 0.218 | 0.641 |
| HPV-58 | 8; 1.6% | 23; 2.8% | 1.989 | 0.158 | 22; 1.9% | 22; 2.7% | 1.426 | 0.232 |
| HPV-59 | 6; 1.2% | 13; 1.6% | 0.333 | 0.564 | 15; 1.3% | 3; 0.4% | 4.525 | ＜0.05 |
| HPV-66 | 3; 0.6% | 8; 1.0% | 0.538 | 0.463 | 9; 0.8% | 5; 0.6% | 0.177 | 0.674 |
| HPV-68 | 1; 0.2% | 10; 1.2% | 3.924 | ＜0.05 | 12; 1.0% | 14; 1.7% | 1.729 | 0.189 |
| ^a^Pearson's Chi-square test was performed to compare the prevalence of HPV infection between Han and Mongols in two regions of Inner Mongolia | | | | | | | | |
| ^b^On account of multi-infection, the number of Hr-HPV pos may less than the sum of each HPV genotype | | | | | | | | |

Fig S1. Age-distribution of Hr-HPV prevalence, stratified by region and ethnicity
